# Identification of Significant Anatomical Variations in the Nose and Anterior Skull Base Using Computed Tomography: A Cross-Sectional Study

**DOI:** 10.7759/cureus.8449

**Published:** 2020-06-05

**Authors:** Naureen Farhan, Syeda Uzma Naqvi, Binish Rasheed, Amjad Sattar, Maria Khan, Anila Rahim, Ghulam Murtaza

**Affiliations:** 1 Diagnostic Radiology, Dow Institute of Radiology, Dow University of Health Sciences, Karachi, PAK; 2 Otolaryngology, Head & Neck Surgery, Dow University of Health Sciences, Karachi, PAK; 3 General Surgery, Patel Hospital, Karachi, PAK

**Keywords:** deviated nasal septum, turbinates, optic nerve, agger nasi, keros classfication, ct scan, paranasal sinuses

## Abstract

Introduction

This study is aimed at the identification of anatomic variations in the nose, paranasal sinuses (PNS), and anterior skull base, which are substantially important to ensure safe and complete endoscopic sinus and skull base surgery.

Materials and methods

This cross-sectional study was conducted at the Dow Institute of Radiology, Dow International Medical College, Dow University Hospital. We included adult patients (i.e., those aged 18 years or older) undergoing a non-contrast CT of the nose and PNS. Two consultant radiologists reviewed the scans on the picture archiving and communication system independently. Any conflict was resolved with consensus. Anatomical variations in the nose, PNS, and anterior skull base of both sides were evaluated.

Results

We reviewed the CT of the PNS of 130 patients with an age of 35.8 ± 14.48 years (mean ± standard deviation). The proportion of men (64/130; 49.2%) and women (66/130; 50.8%) was equal. All patients had one or more variations. The most common abnormality was a deviated nasal septum (DNS), observed in 115 of 130 participants (88.5%) with unilateral occurrence predominant. It was followed by inferior nasal turbinate hypertrophy and agger nasi cells in 76.2% and 67.7% patients, respectively. Optic nerve variation type I (160/260 sinuses; 61.5%) and Keros type II, for olfactory depth (162/260 sinuses; 62.3%), were most common.

Conclusions

Here we report anatomical variations in PNS in all patients of our study; the commonest of all anatomical variations was a DNS. A CT scan is instrumental in surgical planning and patient safety in functional endoscopic sinus surgery.

## Introduction

Skull base surgery is highly complicated and challenging as its working area is around vital structures. The identification of important anatomic variants of paranasal sinuses (PNS) is crucial in the planning of functional endoscopic sinus surgery (FESS) or other skull base surgical procedures. Therefore, preoperative CT scans of PNS are a prerequisite [1].

Radiologists should identify and report these anatomical variations so that the operating surgeon anticipates technical challenges, and the patient can give informed consent [2]. Many of these anatomical variants are causes of sinonasal inflammatory disease, and tailored surgery is required to prevent recurrence [3]. Failure to recognize these variants is associated with a higher rate of surgical complications [4].

Therefore, our study is aimed at the identification of anatomic variations in the nose and PNS, which are substantially important to ensure safe and complete endoscopic sinus and skull base surgery.

## Materials and methods

This cross-sectional study was conducted at the Dow Institute of Radiology, Dow University of Health Sciences. An extensive search was conducted to retrieve the PNS CT scans of the last two years until the required sample size was achieved. Formal approval from the internal review board was taken (IRB-1462/DUHS/approval/2019/111); informed consent was waived as there was no human interaction for this study and only CT scans were reviewed.

CT scans of adult patients (aged 18 years or older) of both sexes were included. Patients with extensive sinonasal inflammatory disease distorting anatomy of nose and PNS; tumors; prior history of trauma or surgical procedures around the nose or PNS; and patients other than those of Pakistani origin were excluded.

Non-contrast CT scans of the nose and PNS were performed as per departmental protocol: 120 kV, 50 mA, pitch 0.533, rotation time 0.5 sec, 3-5 mm axial sections followed by reformation on sagittal and coronal planes, both in soft tissue and bone windows. The scans meeting inclusion and exclusion criteria were selected. Two consultant radiologists (with over nine and seven years’ experience, respectively, after a fellowship in diagnostic radiology) reviewed the scans on the picture archiving and communication system individually; any conflict was resolved by consensus. Anatomical variants of the nose, PNS, and anterior skull base of both sides were recorded in proforma along with the biodata of patients.

The sample size was calculated on World Health Organization (WHO) software for sample size determination in health studies (powered by National University of Singapore); assuming 5.7% of subjects with anatomical variations and 4% bound on error, a sample of 130 participants was required. Data were entered and analysed using IBM SPSS Statistics for Windows, Version 19.0 (IBM Corp., Armonk, NY). Continuous variables were analysed as means ± standard deviations. Categorical variables like anatomical variations and gender were analysed as proportions and percentages while using total participants (n=130) as the denominator except for optic nerve variations and Keros (olfactory depth) classification. The frequency for these two variations was calculated by using total sinuses (n=260) as the denominator.

## Results

We reviewed the CT PNS of 130 patients (total 260 sinuses) with an age of 35.8 ± 14.48 years. The proportion of men (64/130; 49.2%) and women (66/130; 50.8%) was equal. All patients had at least one anatomical variation. The most common variation in PNS was a deviated nasal septum (DNS), observed in 115 of 130 (88.5%) patients (Table 1). It was followed by inferior nasal turbinate hypertrophy and agger nasi (AGN) cells in 99 of 130 (76.2%) and 88 of 130 (67.7%) patients, respectively. We found dehiscence of the optic nerve in sphenoid sinus in 21 of 130 (16.5 %) subjects, which is quite high as reported in former studies.

**Table 1:** Anatomical variations with frequencies calculated for total participants (n=130) ICA, internal carotid artery

Variation	Frequency (n=130)	Side		
		Right	Left	Bilateral
Deviated nasal septum	115 (88.5%)	47 (36.2%)	53 (40.8%)	15 (11.5%)
Turbinate hypertrophy	99 (76.2%)	28 (21.5%)	21 (16.2%)	50 (38.5%)
Paradoxical middle turbinate	16 (12.3%)	9 (6.9%)	4 (3.1%)	3 (2.3%)
Concha bullosa	43 (33.1%)	8 (6.2%)	13 (10%)	22 (16.9%)
Agger nasi cells	88 (67.7%)	6 (4.6%)	6 (4.6%)	76 (58.5%)
Onodi cells	38 (29.2%)	6 (4.6%)	6 (4.6%)	26 (20%)
Haller cells	28 (21.5%)	7 (5.4%)	5 (3.8%)	16 (12.3%)
Maxillary sinus septa	34 (26.2%)	3 (2.3%)	9 (6.9%)	22 (16.9%)
Atelectatic ifundibulum	3 (2.3%)	1 (0.8%)	-	2 (1.5%)
Septal pneumatization	13 (10%)	-	-	-
Clinoid process pneumatization	32 (24.6%)	12 (9.2%)	9 (6.9%)	11 (8.5%)
Pterygoid pneumatization	41 (31.5%	7 (5.4%)	17 (13.1%)	17 (13.1%)
Crista galli pneumatization	11 (8.5%)	-	-	-
Optic nerve dehiscence	21 (16.4%)	8 (7.3%)	6 (5.5%)	4 (3.6%)
ICA dehiscence	2 (1.5%)	-	1 (0.8%)	1 (0.8%)
Sphenoidal septa attached to the ICA	17 (13.1%)	5 (3.8%)	6 (4.6%)	6 (4.6%)
Sphenoidal septa attached to the optic nerve	11 (8.5%)	2 (1.5%)	3 (2.3%)	6 (4.6%)

**Figure 1:**
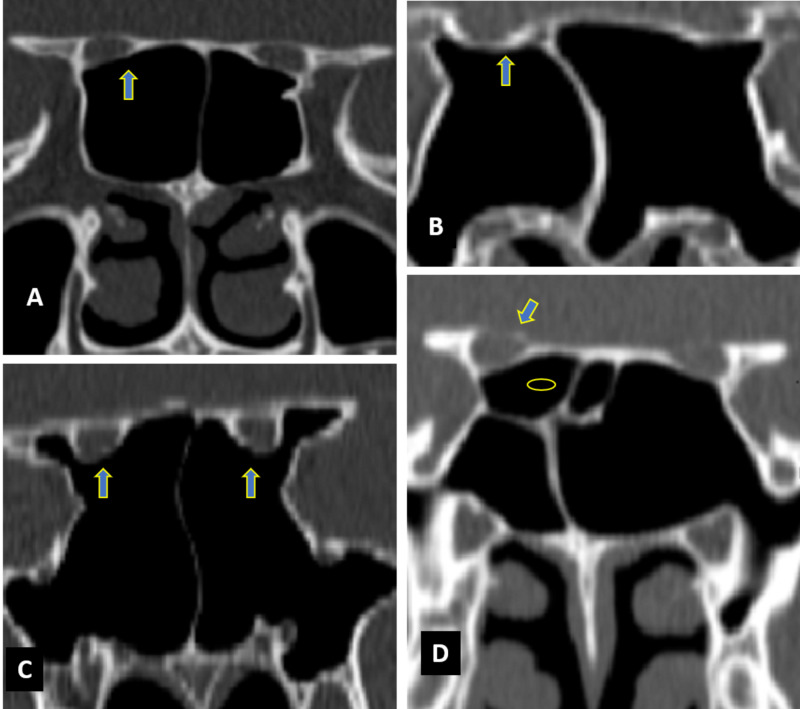
Coronal CT images of paranasal sinuses showing optic nerve variations (arrows) Optic nerve (A) type I bilaterally, (B) type II bilaterally, (C) type III bilaterally, (D) type IV on the right side and type I on the left side (oval indicates posterior ethamoidal sinus).

**Figure 2:**
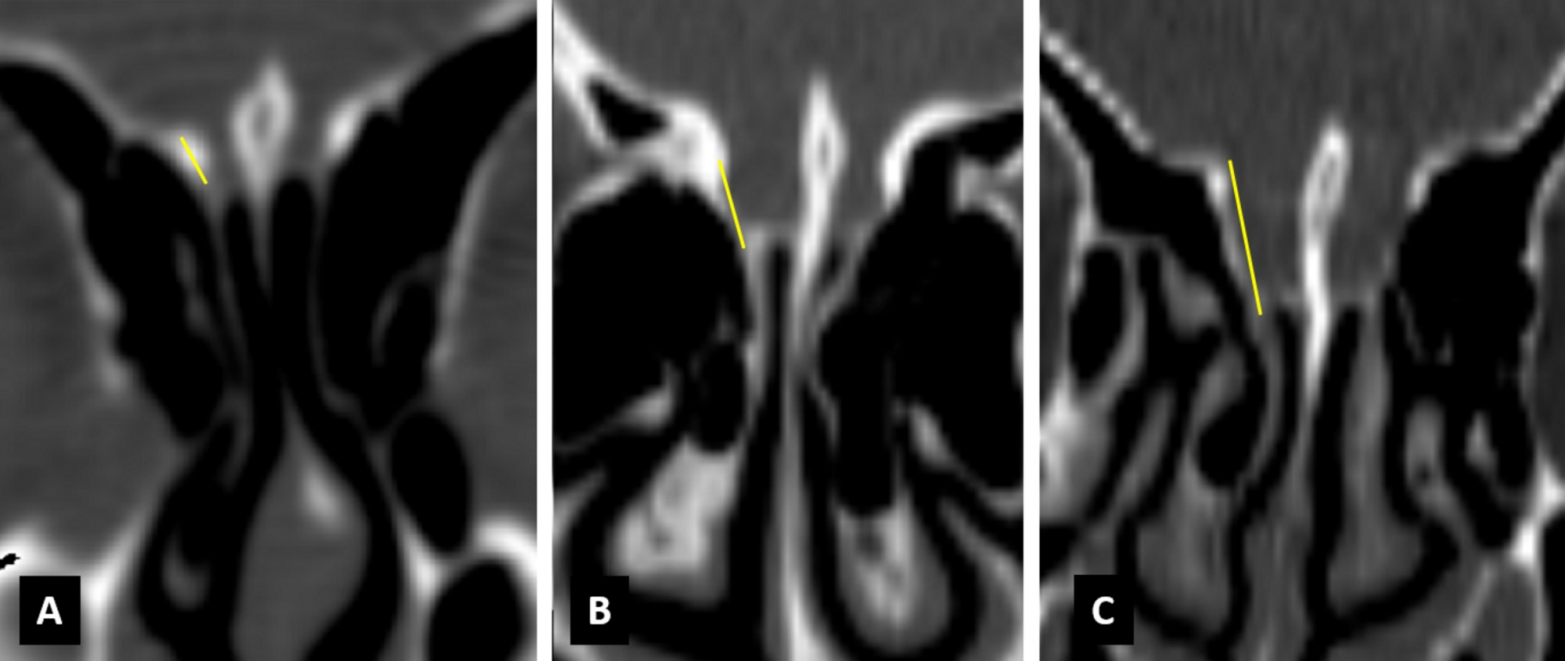
Coronal CT images showing Keros classification Keros (A) type I bilaterally, (B) type II bilaterally, and (C) type III bilaterally.

The frequencies for variations of the optic nerve and olfactory fossa depth by Keros type were calculated for total sinuses (i.e., 260 sinuses) (Figures 1, 2). Optic nerve variation type I was the most common of all, observed in 160 of 260 (61.5%) cases (Table 2). Similarly, Keros type II was the most common variation of all, observed in 162 of 260 (62.3%).

**Table 2:** Anatomical variations with frequencies calculated for total sinuses (n=260)

Variation	Frequency (n=260)	Right side	Left side
Optic nerve variations			
Type I	160 (61.5%)	79 (30.3%)	81 (31.2%)
Type II	58 (22.3%)	31 (11.9%)	27 (10.3%)
Type III	12 (4.6%)	5 (1.9%)	7 (2.6%)
Type IV	30 (11.5%)	13 (5%)	17 (6.5%)
Keros olfactory fossa			
Type I	67 (25.7%)	36 (13.8%)	31 (11.9%)
Type II	162 (62.3%)	80 (30.7%)	82 (31.5%)
Type III	31 (11.9%)	16 (6.1%)	15 (5.7%)

## Discussion

In our study, the most common anatomical variant found was a DNS, identified in 115 (88.5%) subjects. It is much higher than 36% as reported by Beale et al. [5]. However, one study from India revealed 88.2% of patients with DNS among 85 subjects [6].

Concha bullosa (CB) denotes the pneumatization of the middle turbinate. This variant is important as it reduces the nasal patency and induces rhinosinusitis by narrowing ostiomeatal complex. In our study, we observed 43 (33.1%) cases with CB, in which, a bilateral presentation was seen in 22 (16.9%). This is higher than reported in one local study (18.2%) [7]. However, studies conducted in the UK and Italy have reported comparable results (i.e., 28% and 29%, respectively) [8-10].

Paradoxical middle turbinate is the reverse presentation of the middle turbinate with maximum curvature towards the lateral wall of the nose, which leads to the narrowing of ostiomeatal complex. Our data found 16 (12.3%) cases of this anomaly: nine (6.9%) on the right, four (3.1%) on the left, and three (2.3%) identified bilaterally. These results are very close to other studies, which reported from 11% to 14.3% cases [7,9,11-13].

AGN cells are considered the most anterior ethmoid cells. The presence of these cells can obstruct the patency of the frontal recess [5]. We observed AGN cells in 88 (67.7%) subjects, and mostly, they were present on both sides. Alrumaih et al. reported AGN cells in 97.5% of a Saudi population, and all were bilateral [11]. An Indian study reported a 50% prevalence of AGN cells [14].

Onodi cells are the lateral extension of posterior ethmoid cells. These are clinically important because of close proximity to the internal carotid artery (ICA) and optic nerve. In our study, Onodi cells were found in 38 (29.2%) subjects, and 26 (20%) were identified bilaterally. The presence of Onodi cells in other ethnic groups has also been reported (i.e., 28.9% in Arabs, 10% in Turkey, and 25% in Thailand) [11,15,16]. In addition, a previous local study reported around 7.8% cases [7].

**Figure 3:**
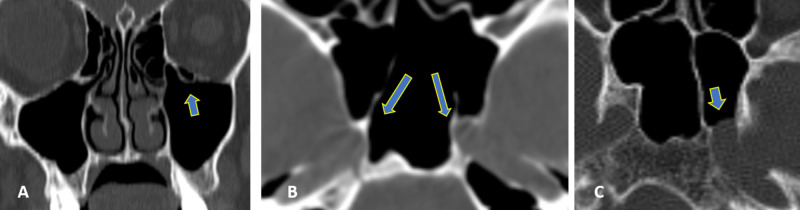
CT images with variations (arrows) (A) Left Haller cells, (B) sphenoid sinus septa attached to bilateral carotid artery canals, and (C) left carotid artery dehiscence.

Haller cells are ethmoidal cells present on the inferomedial orbital wall (Figure 3) [5]. In a study from India, they were reported in 17.5% cases [14]. One local study reported 9.1% cases, while our study identified Haller cells in 28 (21.5%), and 16 (12.3%) were present bilaterally [7].

Pneumatization of the anterior clinond process has been reported in around 6% to 13% of subjects; however, in this study, we found this in 32 (24.6%) subjects [5]. We identified 41 (31.5%) pterygoid processes, 11 (8.5%) crista galli, and 13 (10%) septal pneumatizations. Atelectatic infundibulum was found in three subjects only; these have not been given due attention in the published literature.

The Keros classification for olfactory groove defines depth in accordance with the lateral lamella cribriform plate height, i.e., type I (0-3 mm depth), type II (3.1-7 mm), and type III (>7 mm) (Figure 2). In our study, we found type I in 67 (25.7%), type II in 162 (62.3%), and type III in 31 (11.9%) of the total sinuses (260). Murthy et al. reported Keros type I in 19.5%, type II in 71.5%, and type III in 9% in an Indian population; Sari et al. reported Keros type I in 20.3%, type II in 51.9%, and type III in 27.7% cases in a Turkish population [17,18]. So this corroborates that the most common olfactory groove depth variation is type II, as seen in most of the literature.

Optic nerve variations are classified as four types in accordance with sphenoid and posterior ethmoid sinuses, i.e., type I (adjacent to sphenoid sinus), type II (an optic nerve is indenting on the sphenoid sinus), type III (optic nerve traversing the sphenoid sinus), and type IV (an optic nerve is adjacent to both sphenoid and posterior ethmoid sinuses) (Figure 1). In our study, the most common is type I, found in 160 (61.5%), type II found in 58 (22.3%), type III in 12 (4.6%), and type IV in 30 (11.5%) of a total of 260 sinuses. Delano et al. reported types I, II, III, and IV as 76%, 15%, 6%, and 3%, respectively, which also favor our finding of type I variation being the most common [19].

ICA traverses in a bony canal present in the posterolateral wall of the sphenoid sinus (Figure 3). Deficient bony separation makes ICA bare in the sphenoid sinus, which carries potentially high chances of injury during surgery. In one study from Libya, this anomaly was reported in 30% of cases [20]. Our data showed that two patients (1.5%) had ICA dehiscence: one case of unilateral (left-sided) and one case of bilateral. It is comparable with findings of Lupascu et al. and Unal et al. who reported ICA dehiscence in 2% and 4.7% cases, respectively [21,22].

In our study, we found optic nerve dehiscence in the sphenoid sinus in 21 (16.5 %) subjects. This is much higher than that reported by Lupascu et al. and Unal et al.: 5% and 7%, respectively [21,22].

A variable prevalence of septa of the sphenoid sinus has been reported for the septa of the sphenoid sinus. In our study, we have found a single intersphenoidal septum in 72.3% of subjects. Out of these, 30.2% deviated towards the right, 26.5% towards the left, and 13.7% were found in the midline. Multiple or accessory intrasphenoidal septa were identified in 24.5% of cases and an absent intersphenoidal septum was identified in 3.2% of subjects. Our findings are comparable with most of the studies done in an Asian population [23,24]. Battal et al. identified single intersphenoid septum in 64.3% and multiple intersphenoid septa in 32.1% cases of a Turkish population [25]. Lupascu et al. has found single septum in 38% and multiple or secondary septa in 47% of cases [21].

In our study, we found that sphenoid sinus septa attached to neurovascular structures, such as intersphenoidal or accessory septa, were attached to the ICA in 17 (13.1%) subjects (Figure 3). Of these, five (3.8%) were found on the right, six (4.6 %) were on the left, and six (4.6%) were present bilaterally. These data are somewhat larger than those found by Kazkayasi et al., who reported 5.2% cases, but much lower than Lupascu et al., who reported 68.3% [21,26].

In our study, we found that intersphenoidal or accessory septa attached to the optic nerve in 11 (8.5%) subjects. This is higher than results from Kazkayasi et al., who reported 4.1%, but much lower than Lupascu et al.'s who reported 65% and Unal et al. who reported 27.3% cases [21,22,26].

The strengths of our study are that (1) all the scans were evaluated by two senior radiologists and any disparity was resolved with consensus and (2) a wide range of anatomical variations were reviewed that have not been reported in any single study from our region. The limitation of our study is the lack of clinical outcomes. This is a future directive that the CT scan variations should be followed up for surgical outcomes like iatrogenic injuries, morbidity, and mortality. Preventing these adverse outcomes is the ultimate aim of prescribing CT scan preoperatively.

## Conclusions

This study reports the anatomical variations of the nose, PNS, and anterior skull base on CT scans in a Pakistani population. At least one variation was observed all the patients, and the most common anatomical variation was DNS. There is a difference in some of the variables as optic nerve dehiscence was much higher, contradictory to results reported earlier. Hence, it is of crucial importance for the operating surgeon to know the anatomical variation of the nose and PNS to prepare for technical challenges during surgery.
